# Survivin expression promotes VEGF-induced tumor angiogenesis via PI3K/Akt enhanced β-catenin/Tcf-Lef dependent transcription

**DOI:** 10.1186/1476-4598-13-209

**Published:** 2014-09-09

**Authors:** Jaime G Fernández, Diego A Rodríguez, Manuel Valenzuela, Claudia Calderon, Ulises Urzúa, David Munroe, Carlos Rosas, David Lemus, Natalia Díaz, Mathew C Wright, Lisette Leyton, Julio C Tapia, Andrew FG Quest

**Affiliations:** Laboratorio de Comunicaciones Celulares, Centro de Estudios Moleculares de la Célula (CEMC), Programa de Biología Celular y Molecular, ICBM, Facultad de Medicina, Universidad de Chile, Santiago, Chile; Departamento de Cirugía, Hospital Clínico de la Universidad de Chile, Santiago, Chile; Department of Immunology, St Jude Children’s Research Hospital, Memphis, TN 38105 USA; Programa de Biología Celular y Molecular, ICBM, Facultad de Medicina, Universidad de Chile, Santiago, Chile; Laboratory of Molecular Technology, National Cancer Institute, FCRF, Frederick, MD USA; Programa de Morfología y Biología del Desarrollo, ICBM, Facultad de Medicina, Universidad de Chile, Santiago, Chile; Institute of Cellular Medicine, Newcastle University, Newcastle Upon Tyne, UK; Advanced Center for Chronic Diseases (ACCDiS), Santiago, Chile

**Keywords:** Survivin, Angiogenesis, VEGF, β-catenin, PI3K, Akt

## Abstract

Early in cancer development, tumour cells express vascular endothelial growth factor (VEGF), a secreted molecule that is important in all stages of angiogenesis, an essential process that provides nutrients and oxygen to the nascent tumor and thereby enhances tumor-cell survival and facilitates growth. Survivin, another protein involved in angiogenesis, is strongly expressed in most human cancers, where it promotes tumor survival by reducing apoptosis as well as favoring endothelial cell proliferation and migration. The mechanisms by which cancer cells induce VEGF expression and angiogenesis upon survivin up-regulation remain to be fully established. Since the PI3K/Akt signalling and β-catenin-Tcf/Lef dependent transcription have been implicated in the expression of many cancer-related genes, including survivin and VEGF, we evaluated whether survivin may favor VEGF expression, release from tumor cells and induction of angiogenesis in a PI3K/Akt-β-catenin-Tcf/Lef-dependent manner. Here, we provide evidence linking survivin expression in tumor cells to increased β-catenin protein levels, β-catenin-Tcf/Lef transcriptional activity and expression of several target genes of this pathway, including survivin and VEGF, which accumulates in the culture medium. Alternatively, survivin downregulation reduced β-catenin protein levels and β-catenin-Tcf/Lef transcriptional activity. Also, using inhibitors of PI3K and the expression of dominant negative Akt, we show that survivin acts upstream in an amplification loop to promote VEGF expression. Moreover, survivin knock-down in B16F10 murine melanoma cells diminished the number of blood vessels and reduced VEGF expression in tumors formed in C57BL/6 mice. Finally, in the chick chorioallantoid membrane assay, survivin expression in tumor cells enhanced VEGF liberation and blood vessel formation. Importantly, the presence of neutralizing anti-VEGF antibodies precluded survivin-enhanced angiogenesis in this assay. These findings provide evidence for the existance of a posititve feedback loop connecting survivin expression in tumor cells to PI3K/Akt enhanced β-catenin-Tcf/Lef-dependent transcription followed by secretion of VEGF and angiogenesis.

**Electronic supplementary material:**

The online version of this article (doi:10.1186/1476-4598-13-209) contains supplementary material, which is available to authorized users.

## Background

Angiogenesis is a physiological process characterized by the generation of new blood vessels from preexisting ones. In cancer biology, angiogenesis is required to permit increased delivery of oxygen and nutrients to the nascent tumor [1]. This process, whether physiological or pathological, involves several steps, including release of extracellular factors, endotheliocyte migration, proliferation and formation of new vessels. Amongst all the molecules participating in these events, vascular endothelial growth factor (VEGF) is particularly relevant because it modulates the function of vascular and non-vascular cells [2], and promotes every step of angiogenesis, in both physiological and pathological conditions [3].

In tumors, the inhibitor of apoptosis protein (IAP) survivin has been ascribed highly pleiotropic functions and is associated with tumor progression, metastasis and angiogenesis [4]. Importantly, survivin is overexpressed in essentially all human cancers and generally absent in normal adult tissues [5]. As part of the chromosomal passenger complex, crucial for mitosis, survivin facilitates proliferation [6]. Also, as an IAP, this protein is implicated in the inhibition of apoptosis, although the mechanism by which this is achieved remains a matter of debate. Some possibilities include interaction and stabilization of the anti-apoptotic proteins XIAP [7] or HBXIP [8] and inhibition of pro-apoptotic proteins like *second mitochondria-derived activator of caspases/direct inhibitor of apoptosis binding protein with low pI* (SMAC/DIABLO) [9] or Apoptosis Inducing Factor (AIF) [10]. More recently survivin has been shown to promote invasion and metastasis by enhancing Nuclear Factor kappa-light-chain-enhancer of activated B cells (NF-κB)-dependent transcription of fibronectin [11].

Survivin has also been shown to promote survival of endothelial cells (EC), EC proliferation and angiogenesis, an expected finding given that proliferating EC need to upregulate survivin [12, 13]. Rather intriguingly, down regulation of survivin *in tumor cells* and not in the EC was also shown to reduce angiogenesis in gastric cancer cell lines [14] suggesting that survivin may regulate angiogenesis not only by controlling EC proliferation, but also via mechanisms occurring in the tumor cells that enhance angiogenesis. These findings have been examined in human breast cancer and cervical cancer cell lines [15], and more recently, survivin was shown to favor angiogenesis by enhancing secretion of VEGF [16]. Thus, despite clearly being relevant to the process of angiogenesis, the mechanisms by which survivin expression in tumor cells favors this process remain poorly defined.

Survivin expression is regulated by transcriptional and posttranslational events. Transcription factors implicated in controlling survivin expression include Hypoxia Inducible Factor 1α (HIF-1α, Specificity Protein 1 (Sp-1), NFκB, Signal Transducer and Activator of Transcription 3 (STAT3), Notch and β-catenin-Tcf/Lef [17, 18].

The β-catenin-Tcf/Lef is one of the most studied pathways involved in regulating survivin. Although initially described in *drosophila* development [19, 20], the Wnt/β-catenin signaling pathway was rapidly recognized to play a critical role in human cancer [21, 22]. For instance, the adenomatous poliposis coli (APC) protein is part of the complex involved in β-catenin degradation and APC mutations or deletions are known causes of hereditary colon cancer (*Familial Adenomatous Polyposis coli* patients) [23]. In the absence of Wnt ligands, β-catenin is phosphorylated and targeted for degradation by the multi-protein complex that includes Glycogen Synthase Kinase 3β (GSK-3β), APC, Axin, β-catenin, Casein Kinase 1 and others [24, 25]. When Wnts bind to their receptors, the aforementioned multi-protein complex is disassembled, β-catenin is no longer phosphorylated or degraded, cytoplasmic levels increase and the protein translocates to the nucleus where, together with Tcf/Lef family members, transcription of many genes implicated in development and progression of cancer are increased, including survivin, COX-2, Cyclin D1, Runx-2 and VEGF [26]– [30].

Interestingly, effectors downstream of β-catenin-Tcf/Lef like COX-2 feedback into this pathway and enhance signaling: a study in this respect provided evidence in colon cancer cells showing that prostaglandin E2 (PGE2), a product of COX-2 activity, promotes signaling events that preclude β-catenin degradation [31]. Results from our laboratory have shown that caveolin-1 facilitates the process of β-catenin recruitment to the membrane and thereby precludes β-catenin-Tcf/Lef-dependent transcription of survivin and COX-2 [32, 33]. Rather intriguingly, PGE2 stimulation of colon cancer cells also disrupts the plasma membrane complex containing E-cadherin/Caveolin-1 responsible for sequestration of β-catenin [33]. Thus, outside-in signaling downstream of COX-2 blocks pathways responsible for both the degradation and sequestration of β-catenin, augmenting in this manner βcatenin-Tcf/Lef dependent transcription of several genes important in cancer cells.

Considering the importance of survivin in angiogenesis and the general absence of molecular insight, we examined the possibility that in analogy to the COX2-PGE2 loop, survivin might feedback into the βcatenin/Tcf-Lef pathway and thereby enhance expression of genes important for angiogenesis. Indeed, our studies show that survivin increases β-catenin-Tcf/Lef transcriptional activity, the expression of target genes, such as CyclinD1 or VEGF, vessel density in a mouse model and induces angiogenesis in a VEGF-dependent manner in the chick chorioallantoid membrane model. Importantly, these effects of survivin were shown to be mediated by activation of the PI3K/Akt pathway.

## Results

**Figure 1:**
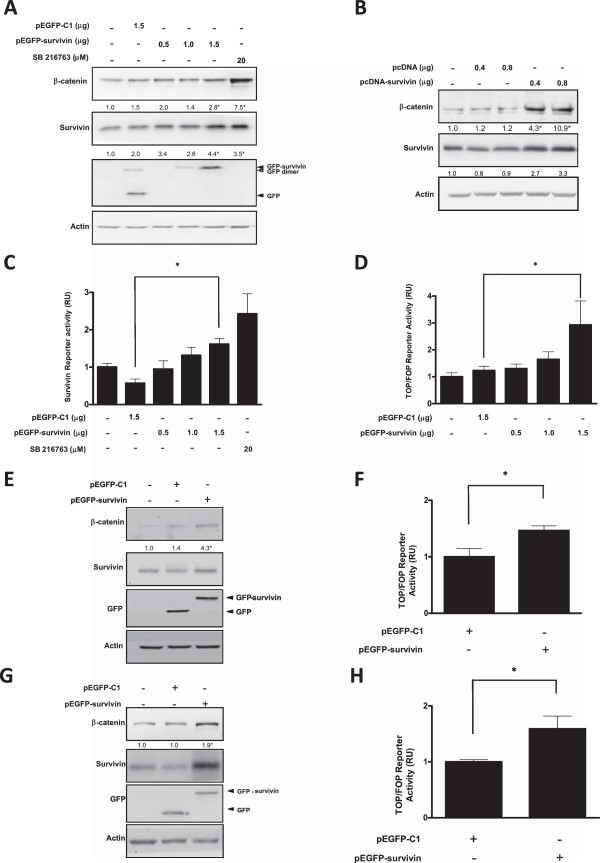
**Survivin expression increased endogenous survivin and β-catenin protein levels, as well as β-catenin/Tcf-Lef dependent transcriptional activity: A,B: HEK293T cells (5×10** ^**5**^ **) were transfected with pEGFP-C1 or pEGFP-survivin (A), pcDNA or pcDNA-survivin (B) or treated with SB216763 (20 μM), a pharmacological inhibitor of GSK-3β.** β-catenin, survivin and actin protein levels were evaluated by western blotting. Protein levels were quantified by scanning densitometric analysis of western blots and normalized to actin. In panel **A** second lane, a band reflecting GFP dimerization is also visible. **C,D**: HEK293T cells (5×10^5^) were seeded in 6-well plates and transiently cotransfected with pEGFP-C1 (1.5 μg) or pEGFP-survivin (0.5-1.5 μg) and additionally with the reporter plasmids pLuc-1710 (intact survivin promoter, 1 μg) pLuc-3M (survivin promoter with 2 TBEs mutated, 1 μg) **(C)**, pTOP-FLASH (three functional TBEs in tandem) or pFOP-FLASH (all TBEs mutated, 1 μg each) **(D)**. Luciferase activity was obtained by calculating the pLuc 1710/pLuc 3M or the pTOP-FLASH/pFOP-FLASH activity ratios for each condition. Data were previously normalized to values for β-galactosidase activity. **E-H**: NIH3T3 **(E,F)** cells (5×10^5^) or MKN45 **(G,H)** cells (5×10^5^) were seeded in 6-well plates and transiently transfected with pEGFP-C1 or pEGFP-survivin (1 μg each). **E**-**G**: β-catenin, survivin, GFP and actin protein levels were evaluated by western bloting. **F-H**: cells were cotransfected with pTOP-FLASH or pFOP-FLASH (1 μg) vectors and luciferase activity was obtained by calculating the pTOP-FLASH/pFOP-FLASH activity ratios for each condition. Data were previously normalized to values for β-galactosidase activity. Numerical data shown are the means ± s.e.m. of results obtained in three independent experiments. Statistically significant differences compared to mock controls are indicated (*p < 0.05).

To assess the effects of survivin on β-catenin protein levels and transcriptional activity, HEK293T cells were transfected with pEGFP-survivin or pcDNA-survivin and their respective empty vector controls. Upon survivin expression, a dose-dependent increase in β-catenin levels was detected together with an increase in endogenous survivin protein that was distinguishable from exogenous GFP-survivin by virtue of its molecular weight (Figure 1A,B). Additionally, as a positive control, β-catenin levels increased in the presence of the GSK3-β inhibitor SB216763 (Figure 1A-B). Because GSK3β activity promotes proteasome-mediated degradation of β-catenin, addition of this inhibitor was expected to increase β-catenin protein levels.Next, the effect of survivin overexpression on β-catenin-Tcf/Lef-dependent transcriptional activity was evaluated using reporter assays for β-catenin-Tcf/Lef activity and a survivin promoter-specific construct. In both cases, a dose dependent increase was detected upon GFP-survivin expression (Figure 1C,D). Subsequently, we evaluated whether the observations in HEK293T cells were also detectable in additional cell lines like mouse fibroblasts (NIH3T3) and human gastric cancer cells (MKN45). For both cell lines, increases in β-catenin protein levels and β-catenin-Tcf/Lef transcriptional activity were observed (Figure 1E-H). However, given the interest here in uncovering a new role for survivin in cancer, we decided to focus our subsequent characterization on the human gastric cancer cell line MKN45 in addition to the human embryonic kidney HEK293T cells.

**Figure 2:**
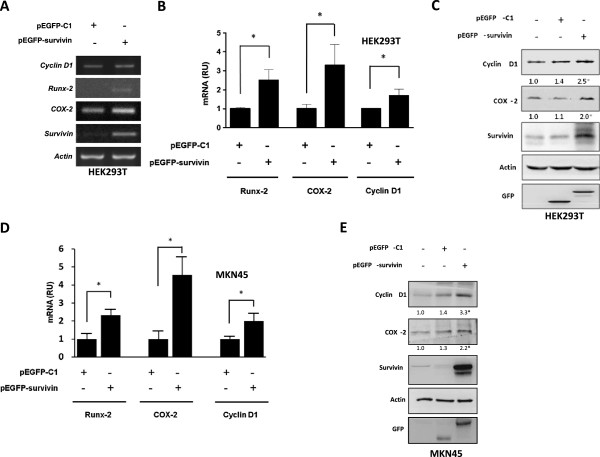
**Survivin expression increased mRNA and protein levels of β-catenin/Tcf-Lef target genes: A,B: HEK293T cells (5 × 10** ^**5**^ **) were seeded in 6-well plates and transfected with pEGFP-C1 or pEGFP-survivin (1.5 μg). A**: After 24 h Cyclin D1, Runx-2, COX-2 and survivin mRNA levels were assessed by semi-quantitative RT-PCR. Actin was used as an internal control **B**: Runx-2,COX-2 and Cyclin D1 mRNA levels were assessed by qPCR. Ribosomal 18S RNA was used as an internal control. Relative Units (RU) reflect normalization to controls. Values are means of results obtained in three independent experiments. Statistically significant differences compared to mock-transfected controls are indicated (* p < 0.05). **C**: Cyclin D1, COX-2 and endogenous survivin levels were evaluated by western blotting. Actin was used as an internal control. Expression of GFP and GFP-survivin in the respective lanes was revealed with a GFP-specific antibody. **D, E**: Wild type MKN45 gastric cancer cells (5×10^5^) were seeded in 6-well plates and transfected with with pEGFP-C1 or pEGFP-survivin (1.5 μg). After 24 h mRNA and protein levels were assessed by qPCR and western blotting, respectively. **D**: Runx-2, COX-2 and cyclin D1 mRNA levels were assessed by qPCR. Ribosomal 18S RNA was used as an internal control. Relative Units (RU) reflect normalization to controls. Values are means of results obtained in three independent experiments. Statistically significant differences compared to mock-transfected controls are indicated (* p < 0.05). **E**: Cyclin D1, COX-2 and endogenous survivin protein levels were evaluated by western blotting, quantified by scanning densitometry of immunoblots and normalized to actin. Numerical data shown are means of results obtained in three independent experiments. Statistically significant differences compared to mock controls are indicated (* p < 0.05).

Accumulation of β-catenin in the nucleus promotes the expression of a wide range of genes related to cancer. In an mRNA-based microarray study, HEK293T cells expressing or not GFP-survivin were compared. A noticeable increase in the relative expression of many Wnt target-genes related to cancer was detectable in this experiment (see Additional file 1: Supplementary information 1). In this context, selected genes associated with cancer were further characterized by RT-PCR. Increases in Runx-2, COX-2 and CyclinD1 mRNA were detected by semi-quantitative RT-PCR (Figure 2A) and by quantitative qPCR (Figure 2B). Moreover, changes in mRNA levels of the respective genes were found to lead to increased expression of the respective proteins as revealed Western blot analysis (Figure 2C). Likewise, in MKN45 gastric cancer cells, GFP-survivin expression also increased significantly mRNA levels of these β-catenin/Tcf-Lef target genes as evaluated by qPCR (Figure 2D). Moreover, an increase in protein levels of endogenous survivin, COX-2, and Cyclin D1 upon GFP-survivin overexpression was detected in MKN-45 cells (Figure 2E) upon analysis by Western Blotting.

**Figure 3:**
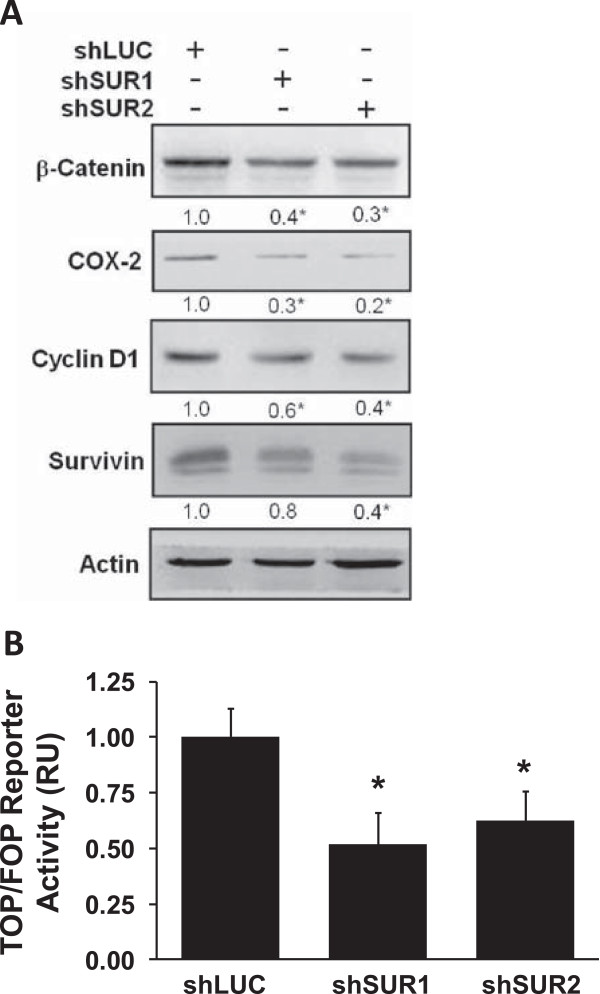
**Survivin downregulation decreased β-catenin protein levels and β-catenin/Tcf-Lef transcriptional activity: A,B: B16F10 cells were stably transfected with 2 different shRNA targeting mouse survivin (shSUR1 and shSUR2) or luciferase (shLUC) as a control.** Batch populations of cells (5×10^5^) were seeded in 6-wells plates **A**: After 24 h, β-catenin, COX-2, Cyclin D1, survivin, and actin protein levels were evaluated by western blotting. Protein levels were quantified by scanning densitometric analysis of western blots and normalized to actin. Numerical data shown are the means of results obtained in three independent experiments. Statistically significant differences compared to shLUC controls are indicated (* p < 0.05). **B**: B16F10 (shLUC, shSUR1 and shSUR2) cells were transiently transfected with the reporter plasmids pTOP-FLASH or pFOP-FLASH (1 μg). Luciferase activity was obtained by calculating the pTOP-FLASH/pFOP-FLASH activity ratios for each condition. Data were previously normalized to values for β-galactosidase activity. Numerical data shown are the means ± s.e.m. of results obtained in three independent experiments. Statistically significant differences between shSUR1-2 and shLUC controls are indicated (* p < 0.05).

To confirm the relevance of these findings, the effect of survivin down-regulation using shRNA technology was evaluated in B16F10 mouse melanoma using virus-mediated cell transduction. This cell line was chosen because they were subsequently employed in tumor formation experiments in syngeneic C57BL6 mice with an intact immune system as previously described by our laboratory [34, 35]. Also, because these cells already have relatively high endogenous levels of survivin as compared with others, further increases in survivin by overexpression had little effect (data not shown). For these reasons, we chose this cell line to implement the opposite approach, namely to downregulate survivin. Indeed, shRNA targeting mouse survivin decreased survivin, β-catenin, COX-2, Cyclin D1 protein levels (Figure 3A). Moreover, β-catenin-Tcf/Lef-dependent transcriptional activity decreased significantly upon survivin knock-down in both shSUR1 and shSUR2 sublines when compared to shLUC control cells (Figure 3B). Alternatively, in ZR-75 human breast cancer cells shRNA-mediated survivin knock-down was evaluated using a commercially available plasmid that permitted selecting populations expressing shRNA against survivin or a scrambled shRNA sequence. Again, both survivin and β-catenin protein levels decreased in cells expressing the survivin-specific sequence, but not in control cells (see Additional file 1: Supplementary information 2).

**Figure 4:**
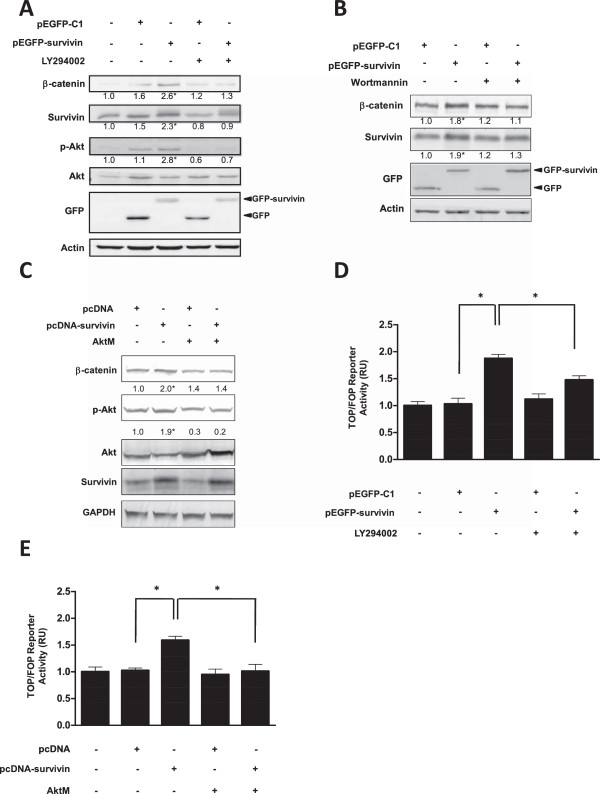
**Survivin effects on β-catenin protein levels and β-catenin/Tcf-Lef transcriptional activity were suppressed by inhibiting PI3K or Akt in HEK293T cells. A,B**: HEK293T cells (5×10^5^) were seeded in 6-well plates and transfected with pEGFP-C1 or pEGFP-survivin (1.5 μg). After transfection, cells were treated with LY294002 (20 μM) or wortmannin (10 nM). After 24 h, β-catenin, survivin, Akt, p-Akt, GFP and actin protein levels were evaluated by western blotting. Protein levels were quantified by scanning densitometric analysis of western blots and normalized to actin. **C**: HEK293T cells were seeded (5×10^5^) in 6-wells plates and co-transfected with pcDNA or pcDNA-survivin (0.8 μg) and a dominant negative form (1 μg) of Akt (AktM). After 24 h, β-catenin, survivin, Akt, p-Akt and GAPDH protein levels were evaluated by western blotting. Protein levels were quantified by scanning densitometric analysis of western blots and normalized to GAPDH. **D**: HEK293T cells were seeded (5×10^5^) in 6-wells plates and co-transfected with pEGFP-C1 or pEGFP-survivin (1.5 μg) and the reporter plasmids pTOP-FLASH or pFOP-FLASH (1 μg). After transfection, cells were treated with LY294002 (20 μM). Luciferase activity was obtained by calculating the pTOP-FLASH/pFOP-FLASH activity ratio for each condition. Data were previously normalized to values for β-galactosidase activity. **E**: HEK293T cells were seeded (5×10^5^) in 6-well plates and transiently cotransfected with pcDNA or pcDNA-survivin (0.8 μg), the reporter plasmids pTOP-FLASH or pFOP-FLASH (1 μg) and in some cases dominant negative Akt (AktM, 1 μg). Luciferase activity was obtained by calculating the pTOP-FLASH/pFOP-FLASH activity ratio for each condition. Data were previously normalized to values for β-galactosidase activity. Numerical data shown are the means ± s.e.m. of results obtained in three independent experiments. Statistically significant differences compared to mock controls are indicated (* p < 0.05).

To explore mechanisms that may explain how survivin affects β-catenin/Tcf-Lef signalling, different pharmacological inhibitors were evaluated. Only PI3K inhibitors were found to suppress the ability of survivin to augment β-catenin levels (see Additional file 1: Supplementary information 3). Specifically, the inhibitor LY294002 was shown to suppress the ability of GFP-survivin to increase endogenous β-catenin and survivin protein levels (Figure 4A). Interestingly, GFP-survivin overexpression also increased the p-Akt/Akt ratio, suggesting activation of the PI3K/Akt pathway, which is known to promote β-catenin-Tcf/Lef dependent transcription (see Discussion). To confirm the role of PI3K, Wortmannin, a well-established PI3K inhibitor, was also shown to suppress GFP-survivin mediated increases in endogenous β-catenin and survivin expression (Figure 4B). Moreover, the effects of expressing a dominant negative Akt variant were evaluated. Here, we used a pcDNA-survivin plasmid because pEGFP-survivin together with the genetic inhibitor decreased cell viability. Dominant negative Akt suppressed the effects of survivin on β-catenin protein levels (Figure 4C). Finally, β-catenin-Tcf/Lef reporter activity was assessed upon LY294002 treatment and expression of dominant negative Akt. In both cases, the ability of survivin to enhance β-catenin-Tcf/Lef transcriptional activity was suppressed (Figure 4D,E).

**Figure 5:**
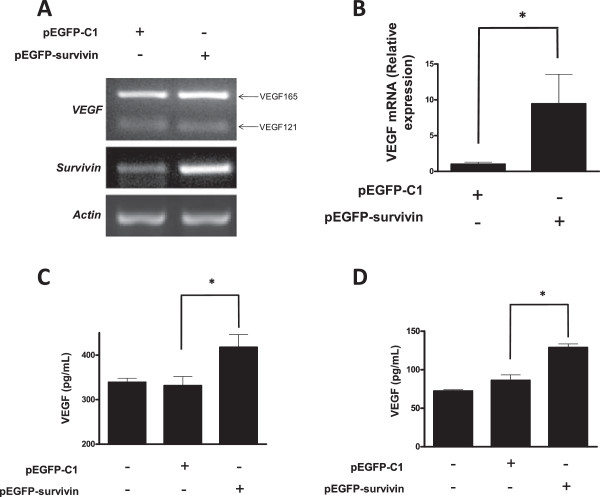
**Survivin expression increased VEGF mRNA and VEGF protein levels in the medium: A,B: HEK293T cells (5×10** ^**5**^ **) were seeded in 6-well plates and transfected with pEGFP-C1 or pEGFP-survivin (1.5 μg). A**: After 24 h, VEGF (VEGF_165_ and VEGF_121_) and survivin mRNA levels were assessed by semi-quantitative RT-PCR. Actin was used as internal control. **B**: Total VEGF mRNA levels were assessed by qPCR. Ribosomal 18S RNA was used as internal control. **C,D** Extracellular VEGF was determined by an ELISA assay of supernatants from HEK293T **(C)** or MKN45 cells **(D)** transfected with pEGFP-C1 or pEGFP-survivin. Numerical data shown are the means ± s.e.m. of total VEGF measured in culture supernatants obtained in three independent experiments. Statistically significant differences compared to mock controls are indicated (* P < 0.05).

To explore the relationship between survivin and angiogenesis, we focused first on evaluating VEGF expression, a target of β-catenin-Tcf/Lef. An increase in VEGF mRNA expression had previously been detected by microarray analysis following GFP-survivin overexpression (see Additional file 1: Supplementary information 1). Hence, changes in VEGF mRNA variants were evaluated upon overexpression of GFP-survivin in HEK293T cells. Indeed, VEGF_165_ increased under these conditions as observed by semiquantitative RT-PCR (Figure 5A) and quantitative PCR (Figure 5B). Moreover, VEGF levels in the culture medium increased upon GFP-survivin overexpression in HEK293T and MKN45 as detected using an ELISA assay (Figure 5C,D).

**Figure 6:**
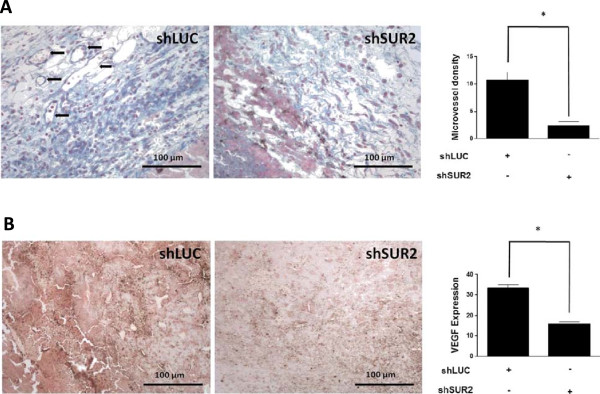
**Angiogenesis and VEGF expression were reduced in tumors derived from survivin-down regulated B16F10 cells: A,B: B16F10 cells stably transfected with shLUC or shSUR2 were used (see Figure** 3 **).** Cells (3 × 10^5^) were subcutaneously injected in 8–10 week-old C57BL6 mice (6 mice per treatment). Once the tumors reached a volume of approximately 1500mm^3^ mice were sacrificed, tumors extracted, measured, divided in halves and formalin-fixed for 24 h. After that samples were paraffin-included, sectioned and then slices were de-paraffined and hydrated. **A**: Samples were stained with arteta, which allows direct visualization of blood vessels. The left panel shows a representative section of a tumor derived from shLUC transfected cells, the center panel, a tumor derived from shSUR2 transfected cells and the right panel, the quantification of average blood vessel density (10 fields per mouse, total 60 fields, * p < 0.05). **B**: samples were analyzed by immunohistochemistry with anti-VEGF specific antibodies. The left panel shows a representative section of a tumor derived from shLUC transfected cells, the center panel, a tumor derived from shSUR2 transfected cells and the right panel depicts the VEGF-Expression Level Score (ELS) obtained as described in Material & Methods for both conditions (10 samples each condition, * p < 0.05). Magnification bar = 100 μm.

Further, the *in vivo* role of survivin in cancer cells was evaluated using cells stably transfected with shLUC and shSUR2 (Figure 3A). C57BL6 mice were subcutaneously injected with these cells and tumors were allowed to grow to the same volume in all cases. Histological analysis revealed that tumours derived from shLUC-transfected cells had more vessels than those formed by shSUR2-transfected cells (Figure 6A photographs). Average blood vessel quantification expressed as microvessel density corroborates these observations (Figure 6A, graph). Also, immunohistochemical analysis with an antibody directed against VEGF revealed decreased VEGF expression in tumours derived from shLUC-transfected cells (Figure 6B, photographs). Quantification of VEGF-specific staining corroborates these findings (Figure 6B, graph).

**Figure 7:**
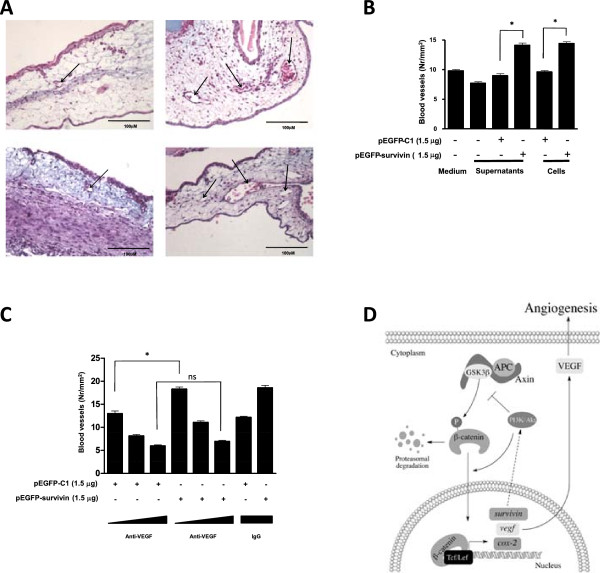
**Survivin expression augmented VEGF-dependent angiogenesis in the chick-CAM assay: A,B: HEK293T cells (2×10** ^**6**^ **) were seeded in 60 mm plates, transfected with pEGFP-C1 or pEGFP-survivin (5μg) and after 48 h supernatants were collected and centrifuged.** Also, cells in suspension were obtained (10^6^/mL). The solutions, fresh medium alone, or media obtained from non-transfected cells or cells in suspension, were pipetted onto chick chorioallantoic membranes (CAM) as described in Materials and Methods. Per condition 3 eggs were employed. A week later CAMs were fixed and stained with hematoxilin-eosin. Three sections per egg were analyzed. **A**: Vessels (arrows) were photographed and counted (10 photographs per section). **B**: The number of vessels per mm^2^ optic field (mean ± s.e.m., n = 3, *: p < 0.05) are depicted. **C**: Supernatants of pEGFP-C1 and pEGFP-survivin obtained as described (A,B), were treated with increasing concentrations of anti-VEGF neutralizing antibodies (0.1, 1, 10 μg/mL). As an IgG control, anti-β3 integrin was used at the highest concentration (10 μg/mL). Numerical data shown are the means ± s.e.m. of results obtained in three independent experiments. Statistically significant differences compared to mock controls are indicated (* = p < 0.05). **D**: Model: β-catenin translocates to the nucleus where it binds Tcf/Lef and induces the expression of target genes (*survivin*, *vegf*, *cox-2* are depicted). Survivin (by unknown mechanisms) favors PI3K/Akt activation, which increases β-catenin-Tcf/Lef transcriptional activity, thereby promoting its own expression (feeding this positive feedback loop), as well as that of *cox-2* and *vegf*. The protein VEGF is secreted to the extracellular compartment where it induces angiogenesis.

Finally, the effect of survivin overexpression was evaluated in an *in-vivo* model of angiogenesis using the chicken chorioallantoic membrane assay (Chick-CAM assay). To this end, either supernatants from HEK293T cells transfected with pEGFP or pEGFP-survivin collected after 48 h or the cells themselves were applied to chorioallantoic membranes and evaluated. Both pEGFP-survivin-transfected HEK293T cells and supernatants from the same cells increased the number of blood vessels in the CAM assays (Figure 7A,B). Importantly, the effect of supernatants from EGFP-survivin expressing cells on angiogenesis was abolished in the presence of specific neutralizing antibodies against VEGF in a specific manner, because treatment with unrelated antibodies (β-3 antibody, negative control) were not able to suppress the GFP-survivin effect (Figure 7C).

## Discussion

Survivin is widely implicated in processes related to tumor development and progression due to its ability to inhibit apoptosis, promote cell cycle progression, favor metastasis and enhance angiogenesis [4]. While the connection between survivin and angiogenesis has been extensively documented, the evidence available so far largely points towards survivin as an enhancer of endothelial cell viability [13]. Here, we provide evidence highlighting a role for survivin in angiogenesis by promoting VEGF expression in tumor cells. Indeed, survivin expression was associated with enhanced β-catenin/Tcf-Lef reporter activity via a PI3K/Akt-dependent mechanism. As a consequence, expression of several target genes including VEGF was enhanced and VEGF accumulated in the medium of tumor cells. Consistent with the notion that survivin dependent release of VEGF is relevant to tumor growth *in vivo*, vascularization of tumors formed by cells with reduced survivin levels was diminished. Moreover, conditioned medium from cells expressing survivin induced angiogenesis in a chick CAM assay and this effect was avoided using VEGF neutralizing antibodies. Thus, survivin is shown here for the first time to enhance VEGF expression in tumor cells via a PI3K/Akt/β-catenin/Tcf-Lef-dependent mechanism and to thereby promote angiogenesis.

Our previous studies revealed that CK2 promoted tumor cell viabillity by enhancing β-catenin/Tcf-Lef-dependent expression of survivin. Moreover, these studies showed that overexpression of survivin alone was sufficient to revert the detrimental effects of CK2 inhibition on cell viability [36]. This was surprising since many β-catenin/Tcf-Lef target genes were affected by CK2 inhibition and suggested that survivin might participate in a loop that feeds back again into the β-catenin/Tcf-Lef pathway. Indeed, our results showed that overexpression of EGFP-survivin increased cytoplasmic β-catenin protein levels and the expression of β-catenin-Tcf/Lef target genes including *COX2*, and *Survivin* itself. Also, downregulation of survivin B16F10 (mouse melanoma) cells reduced cytoplasmic β-catenin, as well as β-catenin-Tcf/Lef-dependent transcriptional activity (Figure 3).

Consistent with the notion that survivin did indeed promote β-catenin/Tcf-Lef–dependent transcription, generic β-catenin/Tcf-Lef reporter activity, as well as activity of a reporter specific for survivin itself increased upon introducing survivin either alone or as a GFP-survivin fusion protein (Figures 1C, D, F, H). Moreover, a DNA microarray analysis revealed increases in the expression of a considerable number of β-catenin-Tcf/lef dependent genes (see Additional file 1: Supplementary information 1), including genes involved in proliferation (Cyclin D1), angiogenesis (VEGF), invasion (MMP-9) and metastasis (CD44). These findings illustrating global changes in gene expression were confirmed in specific cases by RT-PCR and qPCR, such as for Runx-2 and VEGF. Thus, while previous reports in the literature indicate that survivin expression can promote the activation of many signalling pathways [37] none of these have associated survivin expression with enhanced transcription via β-catenin-Tcf/lef, as documented by the experiments shown here.

Survivin is overexpressed in essentially all human cancer cells and expression has not only been associated with the acquisition of several of the so-called tumor cell traits, as defined by Hanahan and Weinberg [38, 39], but also with maintenance of tumor cell viability *in vitro* and *in vivo*. The ability of survivin to do so is often linked to interactions with other proteins and the formation of multi-protein complexes that control proliferation and cell death [40]. More recently, survivin expression was also shown to enhance the metastatic potential of cancer cells by promoting, together with XIAP, NF-kB-dependent transcription and secretion of fibronectin [11]. Hence, these observations provide a more general framework to understanding why survivin expression is augmented in so many different types of human cancers and why expression in those cells is so important for tumor cell survival.

Previously, COX2 was shown to participate in a feed-forward amplification loop involving β-catenin/Tcf-Lef-dependent transcription by generating PGE2, which stimulated EP receptors and favored inactivation of the multi-protein complex that promotes β-catenin degradation [31]. In doing so, a target gene of β-catenin/Tcf-Lef was shown to enhance signaling via the Wnt pathway in a manner involving PI3K/Akt. This we considered an interesting point since a large number of previous studies established a tight relationship between PI3K/Akt and survivin. For instance, activation of the PI3K/Akt pathway favors survivin expression by enhancing NF-kB transcriptional activity [41]. Furthermore, PI3K/Akt also enhances β-catenin-Tcf/lef-dependent transcription by stabilizing β-catenin, either by inhibiting GSK-3β or by directly phosphorylating β-catenin, which favors translocation to the nucleus [42, 43]. Thus, if survivin expression were to connect to β-catenin/Tcf-Lef via PI3K/Akt signaling, an amplification loop that greatly favors tumor cell survival would be the consequence. An initial screen with inhibitors pointed towards PI3K activation as being key to EGFP-survivin-enhanced expression of endogenous survivin (see Additional file 1: Supplementary information 3). Additional experiments using another PI3K inhibitor and over-expression of a dominant negative Akt construct (AktM) indicated that inhibition of this pathway ablated survivin-induced β-catenin stabilization and activation of reporter constructs (Figure 4). Thus, although we cannot completely exclude alternative interpretations, the results presented here point towards the existence of a survivin-PI3K/Akt connection, and most importantly, identify this connection as part of a survivin-mediated tumor cell survival strategy that harnesses β-catenin-Tcf/lef-dependent transcription in the process (see summary Figure 7D).

The fact that PI3K/Akt signalling and survivin are so tightly linked may not come as a surprise, since all are implicated in events that favor cell survival and proliferation. However, the novelty of our current findings resides in showing that the ability of survivin to promote β-catenin/Tcf-Lef-dependent transcription requires PI3K/Akt. How exactly survivin impacts on PI3K/Akt signalling is a question of considerable interest. Survivin is generally thought to modulate cellular processes via specific binding partners. For instance, survivin is implicated in the control of apoptosis by binding to several partners, including XIAP, SMAC-DIABLO, AIF and HBXIP (see introduction). Interestingly, survivin binding to HBXIP together with HBX, reportedly activates PI3K in several cell models. Whether this particular multiprotein survivin complex or others that remain to be discovered are involved in the events we describe here is an intriguing question that needs to be addressed in future experiments.

Angiogenesis is important for clinical progression of disease and patient survival. Moreover, pharmacological targeting of angiogenesis is an effective approach employed in cancer treatment [44]. The process of tumor vascularization is essential to allow solid tumors to grow beyond a minimal size and it has been suggested that the ability of a tumor cell to produce and secrete VEGF is crucial and involves several steps: 1) The liberation of pro-angiogenic factors from tumor cells, including VEGF; 2) Changes in the morphology of endotheliocytes; 3) Liberation of proteolytic enzymes that degrade the basal lamina; 4) Migration and formation of tubular structures; 5) Proliferation of endotheliocytes and 6) Differentiation into capillaries [2]. As mentioned, VEGF is important in this sequence because it participates in most steps, except the first, by acting on tyrosine kinase receptors of the VEGFR family (VEGFR1 and VEGFR2). Upon activation, VEGFR1 and VEGFR2 promote survival and proliferation, as well as inhibit apoptosis of endothelial cells [3], all known functions of survivin. Consistent with this paradigm, survivin has thus far largely been attributed a role in angiogenesis as a participant downstream of VEGFR signalling (see introduction). In this study, we specifically provide evidence indicative of a novel role for survivin in promoting the production of VEGF in tumor cells via a mechanism involving PI3K/Akt (see Figure 7D).

## Conclusions

We describe here for the first time that survivin enhances the expression of a considerable number of genes by augmenting β-catenin-Tcf/Lef transcriptional activity. This is achieved in a manner dependent on PI3K/Akt signalling in the same cells. In doing so, survivin contributes to the formation of a positive feedback circuit in which survivin increases β-catenin-Tcf/Lef transcriptional activity and this in turn favors expression of target genes and the acquisition of traits commonly associated with tumor development, survival and progression. Growth of solid tumors requires angiogenesis and survivin has been implicated in this process largely as an element that functions downstream of VEGFR signalling. Our current studies show that survivin induces VEGF transcription, expression and accumulation in conditioned media and favors angiogenesis in a VEGF-dependent manner (see Figure 7D).

## Methods

### Materials

Monoclonal anti β-catenin and anti-COX-2 antibodies were from Transduction Laboratories (Lexington, KY). Rabbit polyclonal anti-human survivin and anti-actin antibodies were from R&D Systems (Minneapolis, MN) and Sigma (St. Louis, MO), respectively. Polyclonal rabbit anti-GFP and anti-Cyclin D1 antibodies were from Santa Cruz Biotechnology (Santa Cruz, CA). Polyclonal anti-Akt, anti-p-Akt, and monoclonal anti-GAPDH were from Cell Signaling. Monoclonal anti-VEGF antibody was from R&D Systems. Goat anti-rabbit IgG and anti-mouse IgG antibodies coupled to horseradish peroxidase (HRP) were from Bio-Rad Laboratories (Hercules, CA) and Sigma, respectively. EZ-ECL Chemiluminescence Substrate was from Biological Industries (Kibbutz Beit Haemek, Israel). Superfect Reagent and the Plasmid Midi Kit were from Qiagen (Valencia, CA). TriZOL reagent was from Invitrogen (Carlsbad, CA). AMV reverse transcriptase (AMV RT) and Taq DNA polymerase were from Promega (Madison, WI). Cell medium and antibiotics were from Invitrogen-BRL (Paisley, Scotland, United Kingdom). Fetal bovine serum (FBS) was from Hyclone (Logan, UT). Luciferin was purchased from United States Biological (Swampscott, MA). Inhibitors SB-216763 and wortmannin were purchased from Sigma and the inhibitor 2-morpholin-4-yl-8-phenylchromen-4-one (LY294002) was from Calbiochem (San Diego, CA).

### Cell culture and transfections

HEK293T and NIH3T3 cells were cultured in DMEM, MKN-45 and B16-F10 cells in RPMI medium. In all cases media were supplemented with 10% FBS and antibiotics (10,000 U/ml penicillin and 10 mg/ml streptomycin). HEK293T, NIH3T3, MKN-45 and B16-F10 were transfected with Lipofectamine 2000 according to the manufacturer’s instructions. After 3 h, the medium was diluted with 1 mL of medium together with inhibitors when used. Transfection efficiency was checked by epifluorescence microscopy 24 h after transfection. Cells were then harvested, centrifuged and stored at −80°C.

### Western blotting

Cell extracts were prepared as previously described [45], separated (50–80 μg total protein per lane) by SDS-PAGE on 12% acrylamide minigels (Bio-Rad Laboratories), and transferred to nitrocellulose as described previously [46]. Blots were blocked with 5% milk in 0.1% Tween-TBS and then probed with anti-β-actin (1:5000), anti-COX-2 (1:500), anti-β-catenin (1:1000), anti-Cyclin D1 (1:2000), anti Akt (1:1000), anti p-Akt (1:1000), anti-GAPDH (1:2000) or anti-survivin (1:3000) antibodies. Bound antibodies were detected with HRP-conjugated secondary antibodies and the EZ-ECL system.

### Reporter assays

For β-catenin-Tcf/Lef and survivin promoter reporter assays HEK293T, NIH3T3, MKN-45 and B16F10 cells were transfected with 1 μg of each plasmid: pTOP-FLASH (Tcf/Lef reporter), pFOP-FLASH (mutated Tcf/Lef binding site), pLuc-1710 (survivin promoter) or pLuc420–3M (mutated Tcf/Lef binding site). After transfection (24 h), cells were lysed, luciferase activity was quantified and standardized as described previously [33].

### Analysis of mRNA: RT-PCR and qPCR

#### RT-PCR

Total RNA was isolated with TriZOL™ following instructions provided by manufacturer. RNA samples were spectrophotometrically quantified, characterized by electrophoresis in 1% agarose gels and then used as templates to generate cDNA under standard conditions in the presence of DNAase to eliminate any traces of genomic DNA. Specific PCR products were generated using the following primers: COX-2: sense primer 5′-TTCAAATGAGATTGTGGGAAAATTGCT-3′ and anti-sense primer 5′-AGATCATCTCTGCCTGAGTATCTT-3′; survivin: sense primer 5′-CCGACGTTGCCCCCTGC-3′ and anti-sense primer 5′-TCGATGGCACGGCGCAC-3′; Runx-2: sense primer 5′-CAGTTCCCAAGCATTTCATCC-3′ and anti-sense primer 5′-TCAATATGGTCGCCAAACAG-3′; Cyclin D1: sense primer 5′-ACCTGAGGAGCCCCAACAA-3′ and anti-sense primer 5′-TCTGCTCCTGGCAGGCC-3′; VEGF: sense primer 5′-AGGCCAGCACATAGGAGAGA-3′ and antisense primer 5′-ACCGCCTCGGCTTGTCACAT-3′; actin: sense primer 5′-AAATCGTGCGTGACATTAAGC-3′ and anti-sense primer, 5′-CCGATCCACACGGAGTACTT-3′; and 18S rRNA housekeeping gene: sense primer 5′-TCAAGAACGAAAGTCGGAGG-3′ and anti-sense primer 5′-GGACATCTAAGGGCATCACA-3′.

All reaction products were analyzed after 25–30 amplification cycles, each of which involved consecutive 1-min steps at 94, 55–60, and 72°C. Survivin and COX-2 levels were normalized to actin RNA in semi-quantitative RT-PCR studies.

#### Real-Time quantitative PCR

The results obtained by semi-quantitative studies were confirmed by real-time quantitative PCR (qPCR) analysis with the brilliant SYBR green qPCR kit (Stratagene, La Jolla, CA). The PCR reactions were carried out using a Chromo-4 real-time PCR detection system (Bio-Rad Laboratories) and thermo cycler conditions following suggestions of the manufacturer. The relative gene expression levels were calculated using the 2ΔΔCT method [47]. COX-2, Runx-2 and VEGF levels were normalized to RNA of the 18S rRNA housekeeping gene. All data were expressed relative to values obtained for mock-transfected cells (value = 1).

### shRNA knock-down of survivin expression

#### B16F10 cells

The oligonucleotide containing shRNA candidates for mouse survivin #1,GAAGAACTAACCGTCAGTGAA and #2, CCTACCGAGAACGAGCCTGAT or control shRNA for Luciferase CGCTGAGTACTTCGAAATGTC were prepared as previously described [48]. Post-transfection (48 h), media containing lentivirus were filtered through a 0.45 μm pore and used to transduce B16F10 cells in the presence of 8 μg/ml polybreen. After 24 h cells were selected with puromycin (2μg/ml) for seven days and expression was monitored by Western blotting. Plasmids encoding the envelope protein VSV-γ (pHCMV-G), the packaging plasmid p∆8.9 (pCMV∆R8.9) and pLKO.1 plasmids containing shRNA for survivin and control plasmid containing shRNA for Luciferase (shLuc) were provided by Dr. Claudio Hetz (Universidad de Chile, Santiago, Chile).

#### Quantification of VEGF levels

VEGF extracellular protein levels were determined in supernatants from transfected HEK293T or MKN-45 cells. Supernatants were evaluated using the Quantikine VEGF-ELISA assay (R&D Systems).

#### Mouse melanoma tumor angiogenesis model

C57BL6 8–12 week-old female mice were used. *They* were obtained from *Instituto de Salud Pública* and were kept in the animal facility (*Bioterio*) of the Faculty of Medicine (University of Chile). Protocols to work with these animals were approved by local bioethical committee (University of Chile, Faculty of Medicine) in 2008 for the FONDECYT (National Chilean research agency) research project (#1090071) of Dr. Andrew Quest. Mice (12 animals total, 6 animals per group) were subcutaneously injected with 300.000 B16F10 cells. Roughly two weeks after the injection palpable tumors became detectable and were measured daily. When tumors reached the ethically permitted maximum (2500 mm^3^), mice were sacrificed. Tumors were extracted, divided and then fixed in 10% buffered formalin. After 48 h in fixation solution, they were processed to obtain sections of 5 μm and microvessel density or VEGF were evaluated. Microvessel density quantification: Samples were stained with arteta to improve endotheliocyte visualisation and blood vessels were counted by a trained technician who was unaware of sample identity as described previously [49]. VEGF detection: Histological sections were treated with 3% Peroxide Hydrogen in methanol for 10 minutes and incubated for 30 minutes in Dako Target Retrieval (Dako, CA). After washing with PBS, sections were incubated with Anti-VEGF_165_ Polyclonal Antibody (Millipore™, 1:100), developed according to instructions provided with the Histomouse MAX-AEC Broad Spectrum™ Kit (Invitrogen, Camarillo, CA), counterstained with Haematoxylin and mounted with Clearmount™ (Invitrogen). Using identical microscope and camera settings, five digital images per sample were taken to accurately reflect the overall staining. Immunochemical staining for VEGF from all images was analyzed using the commercially available Image-Pro Plus v. 4.5.029 software (Media Cybernetics, USA). A color file was created that exactly selected the hue, saturation and intensity reflecting protein expression levels. This color file defined the range of the signal and was applied to all samples. The Expression Level Score (ELS%) was determined based on the Mean Density of VEGF-specific staining, defined by the color file, per area evaluated.

#### Chick-CAM assay

Fertilized eggs from White Legorn hens (*Gallus gallus*) were used as described previously [50], all protocols approved by the local ethics committee as stated prviously. Eggs were purchased from the Public Health Institute of Chile, incubated in animals facility of the Faculty of Medicine at 25°C for 24 h, marked at the embryonic pole (apex) and incubated at 37°C for another 72 h. Then a small hole (1 mm) was drilled into the acute pole to extract albumin and thereby avoid adherence of the embryo to the upper cortex. Subsequently, a larger opening (2×1 cm) was created at the embryonic pole and sealed with Saran-wrap. A week later, the plastic cover was removed and a 5 mm diameter methylcellulose filter was placed on the chorioallantoic membrane, and 10 μL of sample (media with or without cells) was added to the filter. Samples included either 3×10^4^ HEK293T cells (transfected with survivin 48 h before the experiment) or supernatants from the same transfected cells. In neutralizing experiments, either anti-VEGF or anti-β1-integrin antibodies were added and mixed with the media 20 min before application to the filters. After 3 days, CAMs were removed from the eggs, fixed in 4% p-formaldehyde, then dehydrated, paraffin embedded and stained with hematoxilin-eosin. Blood vessels were counted manually by a trained technician who was unaware of sample identities.

### Statistical analysis

Results were statistically compared using paired student’s t test. All data were from 3 or more independent experiments. p values (two-tailed) < 0.05, was considered significant.

## Electronic supplementary material

Additional file 1: **Supplementary material.** (DOCX 112 KB)

## References

[1] Folkman J (1971). Tumor angiogenesis: therapeutic implications. N Engl J Med.

[2] Fox SB, Gatter KC, Harris AL (1996). Tumour angiogenesis. J Pathol.

[3] Hicklin DJ, Ellis LM (2005). Role of the vascular endothelial growth factor pathway in tumor growth and angiogenesis. J Clin Oncol.

[4] Altieri DC (2008). Survivin, cancer networks and pathway-directed drug discovery. Nat Rev Cancer.

[5] Kelly RJ, Lopez-Chavez A, Citrin D, Janik JE, Morris JC (2011). Impacting tumor cell-fate by targeting the inhibitor of apoptosis protein survivin. Mol Cancer.

[6] Terada Y (2001). Role of chromosomal passenger complex in chromosome segregation and cytokinesis. Cell Struct Funct.

[7] Dohi T, Okada K, Xia F, Wilford CE, Samuel T, Welsh K, Marusawa H, Zou H, Armstrong R, Matsuzawa S, Salvesen GS, Reed JC, Altieri DC (2004). An IAP-IAP complex inhibits apoptosis. J Biol Chem.

[8] Marusawa H, Matsuzawa S, Welsh K, Zou H, Armstrong R, Tamm I, Reed JC (2003). HBXIP functions as a cofactor of survivin in apoptosis suppression. EMBO J.

[9] Song Z, Yao X, Wu M (2003). Direct interaction between survivin and Smac/DIABLO is essential for the anti-apoptotic activity of survivin during taxol-induced apoptosis. J Biol Chem.

[10] Liu T, Brouha B, Grossman D (2004). Rapid induction of mitochondrial events and caspase-independent apoptosis in Survivin-targeted melanoma cells. Oncogene.

[11] Mehrotra S, Languino LR, Raskett CM, Mercurio AM, Dohi T, Altieri DC (2010). IAP regulation of metastasis. Cancer Cell.

[12] Tran J, Master Z, Yu JL, Rak J, Dumont DJ, Kerbel RS (2002). A role for survivin in chemoresistance of endothelial cells mediated by VEGF. Proc Natl Acad Sci U S A.

[13] Botto S, Streblow DN, DeFilippis V, White L, Kreklywich CN, Smith PP, Caposio P (2011). IL-6 in human cytomegalovirus secretome promotes angiogenesis and survival of endothelial cells through the stimulation of survivin. Blood.

[14] Tu SP, Jiang XH, Lin MC, Cui JT, Yang Y, Lum CT, Zou B, Zhu YB, Jiang SH, Wong WM, Chan AO, Yuen MF, Lam SK, Kung HF, Wong BC (2003). Suppression of survivin expression inhibits in vivo tumorigenicity and angiogenesis in gastric cancer. Cancer Res.

[15] Li QX, Zhao J, Liu JY, Jia LT, Huang HY, Xu YM, Zhang Y, Zhang R, Wang CJ, Yao LB, Chen SY, Yang AG (2006). Survivin stable knockdown by siRNA inhibits tumor cell growth and angiogenesis in breast and cervical cancers. Cancer Biol Ther.

[16] Wang P, Zhen H, Zhang J, Zhang W, Zhang R, Cheng X, Guo G, Mao X, Wang J, Zhang X (2012). Survivin promotes glioma angiogenesis through vascular endothelial growth factor and basic fibroblast growth factor in vitro and in vivo. Mol Carcinog.

[17] Guha M, Altieri DC (2009). Survivin as a global target of intrinsic tumor suppression networks. Cell Cycle.

[18] Lladser A, Sanhueza C, Kiessling R, Quest AF (2011). Is survivin the potential Achilles’ heel of cancer?. Adv Cancer Res.

[19] Riggleman B, Schedl P, Wieschaus E (1990). Spatial expression of the Drosophila segment polarity gene armadillo is posttranscriptionally regulated by wingless. Cell.

[20] McCrea PD, Turck CW, Gumbiner B (1991). A homolog of the armadillo protein in Drosophila (plakoglobin) associated with E-cadherin. Science.

[21] Kraus C, Liehr T, Hulsken J, Behrens J, Birchmeier W, Grzeschik KH, Ballhausen WG (1994). Localization of the human beta-catenin gene (CTNNB1) to 3p21: a region implicated in tumor development. Genomics.

[22] Resnik E (1997). beta-Catenin–one player, two games. Nat Genet.

[23] Kinzler KW, Nilbert MC, Su LK, Vogelstein B, Bryan TM, Levy DB, Smith KJ, Preisinger AC, Hedge P, McKechnie D, Finniear R, Markham A, Groffen J, Boguski M, Altschul S, Horii A, Ando H, Miyoshi Y, Miki Y, Nishisho I, Nakamura Y (1991). Identification of FAP locus genes from chromosome 5q21. Science.

[24] Kimelman D, Xu W (2006). beta-catenin destruction complex: insights and questions from a structural perspective. Oncogene.

[25] Fodde R, Brabletz T (2007). Wnt/beta-catenin signaling in cancer stemness and malignant behavior. Curr Opin Cell Biol.

[26] Zhang T, Otevrel T, Gao Z, Ehrlich SM, Fields JZ, Boman BM (2001). Evidence that APC regulates survivin expression: a possible mechanism contributing to the stem cell origin of colon cancer. Cancer Res.

[27] Howe LR, Subbaramaiah K, Chung WJ, Dannenberg AJ, Brown AM (1999). Transcriptional activation of cyclooxygenase-2 in Wnt-1-transformed mouse mammary epithelial cells. Cancer Res.

[28] Tetsu O, McCormick F (1999). Beta-catenin regulates expression of cyclin D1 in colon carcinoma cells. Nature.

[29] Dong YF, Soung do Y, Schwarz EM, O’Keefe RJ, Drissi H (2006). Wnt induction of chondrocyte hypertrophy through the Runx2 transcription factor. J Cell Physiol.

[30] Zhang X, Gaspard JP, Chung DC (2001). Regulation of vascular endothelial growth factor by the Wnt and K-ras pathways in colonic neoplasia. Cancer Res.

[31] Castellone MD, Teramoto H, Williams BO, Druey KM, Gutkind JS (2005). Prostaglandin E2 promotes colon cancer cell growth through a Gs-axin-beta-catenin signaling axis. Science.

[32] Torres VA, Tapia JC, Rodriguez DA, Parraga M, Lisboa P, Montoya M, Leyton L, Quest AF (2006). Caveolin-1 controls cell proliferation and cell death by suppressing expression of the inhibitor of apoptosis protein survivin. J Cell Sci.

[33] Rodriguez DA, Tapia JC, Fernandez JG, Torres VA, Munoz N, Galleguillos D, Leyton L, Quest AF (2009). Caveolin-1-mediated suppression of cyclooxygenase-2 via a beta-catenin-Tcf/Lef-dependent transcriptional mechanism reduced prostaglandin E2 production and survivin expression. Mol Biol Cell.

[34] Lobos-Gonzalez L, Aguilar L, Diaz J, Diaz N, Urra H, Torres VA, Silva V, Fitzpatrick C, Lladser A, Hoek KS, Leyton L, Quest AF (2013). E-cadherin determines Caveolin-1 tumor suppression or metastasis enhancing function in melanoma cells. Pigment Cell Melanoma Res.

[35] Lobos-Gonzalez L, Aguilar-Guzman L, Fernandez JG, Munoz N, Hossain M, Bieneck S, Silva V, Burzio V, Sviderskaya EV, Bennett DC, Leyton L, Quest AF (2014). Caveolin-1 is a risk factor for postsurgery metastasis in preclinical melanoma models. Melanoma Res.

[36] Tapia JC, Torres VA, Rodriguez DA, Leyton L, Quest AF (2006). Casein kinase 2 (CK2) increases survivin expression via enhanced beta-catenin-T cell factor/lymphoid enhancer binding factor-dependent transcription. Proc Natl Acad Sci U S A.

[37] Kanwar JR, Kamalapuram SK, Kanwar RK (2011). Targeting survivin in cancer: the cell-signalling perspective. Drug Discov Today.

[38] Hanahan D, Weinberg RA (2000). The hallmarks of cancer. Cell.

[39] Hanahan D, Weinberg RA (2011). Hallmarks of cancer: the next generation. Cell.

[40] Altieri DC (2006). The case for survivin as a regulator of microtubule dynamics and cell-death decisions. Curr Opin Cell Biol.

[41] Li W, Wang H, Kuang CY, Zhu JK, Yu Y, Qin ZX, Liu J, Huang L (2012). An essential role for the Id1/PI3K/Akt/NFkB/survivin signalling pathway in promoting the proliferation of endothelial progenitor cells in vitro. Mol Cell Biochem.

[42] Cross DA, Alessi DR, Cohen P, Andjelkovich M, Hemmings BA (1995). Inhibition of glycogen synthase kinase-3 by insulin mediated by protein kinase B. Nature.

[43] Fang D, Hawke D, Zheng Y, Xia Y, Meisenhelder J, Nika H, Mills GB, Kobayashi R, Hunter T, Lu Z (2007). Phosphorylation of beta-catenin by AKT promotes beta-catenin transcriptional activity. J Biol Chem.

[44] Hurwitz H, Fehrenbacher L, Novotny W, Cartwright T, Hainsworth J, Heim W, Berlin J, Baron A, Griffing S, Holmgren E, Ferrara N, Fyfe G, Rogers B, Ross R, Kabbinavar F (2004). Bevacizumab plus irinotecan, fluorouracil, and leucovorin for metastatic colorectal cancer. N Engl J Med.

[45] Felley-Bosco E, Bender FC, Courjault-Gautier F, Bron C, Quest AF (2000). Caveolin-1 down-regulates inducible nitric oxide synthase via the proteasome pathway in human colon carcinoma cells. Proc Natl Acad Sci U S A.

[46] Bender FC, Reymond MA, Bron C, Quest AF (2000). Caveolin-1 levels are down-regulated in human colon tumors, and ectopic expression of caveolin-1 in colon carcinoma cell lines reduces cell tumorigenicity. Cancer Res.

[47] Livak KJ, Schmittgen TD (2001). Analysis of relative gene expression data using real-time quantitative PCR and the 2(−Delta Delta C(T)) Method. Methods.

[48] Urra H, Torres VA, Ortiz RJ, Lobos L, Diaz MI, Diaz N, Hartel S, Leyton L, Quest AF (2012). Caveolin-1-enhanced motility and focal adhesion turnover require tyrosine-14 but not accumulation to the rear in metastatic cancer cells. PLoS One.

[49] Garrido O, Letelier R, Rosas C, Fuenzalida M, Ferreira A, Lemus D (2010). Betamethasone inhibits tumor development, microvessel density and prolongs survival in mice with a multiresistant adenocarcinoma TA3. Biol Res.

[50] Sinning M, Letelier R, Rosas C, Fuenzalida M, Lemus D (2012). Angiogenic potential of the cerebrospinal fluid (CSF) of patients with high-grade gliomas measured with the chick embryo chorioallantoic membrane assay (CAM). Biol Res.

