# Supplementary material for: Survivin expression promotes VEGF-induced tumor angiogenesis via PI3K/Akt enhanced β-catenin/Tcf-Lef dependent transcription
Source: Mol Cancer. 2014 Sep 9;13:209. doi: 10.1186/1476-4598-13-209 (PMC4177250; doi:10.1186/1476-4598-13-209)
Supplement: Supplementary file 1 — Additional file 1: Supplementary material. (DOCX 112 KB) [file 12943_2014_1418_MOESM1_ESM.docx]

**Additional file 1:**

**Supplementary information 1: Method:** HEK293T cells (2x10^6^) were seeded in 6-well plates. Then, 24 h later, cells were transiently transfected with pEGFP-C1 or pEGFP-survivin (5μg) using Lipofectamine 2000 as described by the manufacturer. After another 24 h, cells were harvested, centrifuged and stored at -80°C. Total RNA was isolated with the reagent TriZOL™ following instructions provided by manufacturer. To label cDNA, 20 μg of total RNA was reverse transcribed in the presence of Alexa-647 or Alexa-555-dUTPs. Further details on fluorescent cDNA labeling, hybridization and washes have been described elsewhere (Urzua et al., 2006). A dye-swap microarray experiment was conducted using Whole genome HEEBO (Human Exonic Evidence Based Oligonucleotide) microarrays (Microarrays Inc, Huntsville, AL). Microarrays were scanned at 10 μm resolution in a ScanArray Lite (Perkin Elmer, CA) fluorescence scanner. Scanned images were saved in TIFF format and then extracted with the GenePix Pro III. Green/red and red/green ratios were obtained for every gene and then were separated in 3 categories depending on the magnitude of relative expression referred to as: “increased”, “no change” and “repressed”. **Result:** The relative expression patterns (GFP control as 1) were evaluated by microarray analysis for Wnt pathway target genes upon GFP-Survivin overexpression. For 12 of the genes evaluated, no change was observed in the presence of survivin, while for 10 genes survivin-enhanced expression was detected. Only 3 target genes of the Wnt pathway were repressed in the presence of survivin; of these 2 (SOX-9 and P16ink16) are known to be *down regulated* by the Wnt pathway.


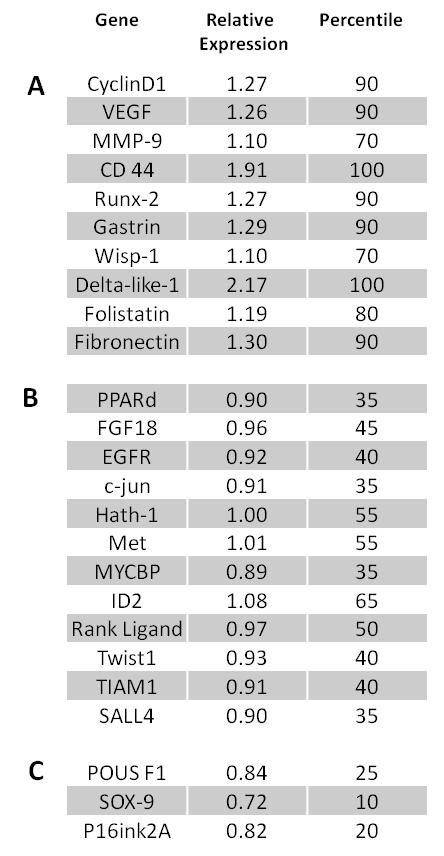


**Table 1: Changes in Wnt pathway target genes induced by GFP-survivin expression.** Microarray analysis was employed to evaluate changes in Wnt target genes expression. **A:** Genes with increased expression (Percentile 70-100), **B:** Genes that didn’t change (Percentile 35-65) **C:** Genes that were repressed (Percentile 0-30). (The complete microarray dataset is available from the Gene Expression
Omnibus (GEO; <http://www.ncbi.nlm.nih.gov/geo/>) repository, under the code
GSE43817)

**Supplementary information 2: Method:** ZR-75 cells were cultured in DMEM-F12 medium supplemented with 10% FBS and antibiotics (10,000 U/ml penicillin and 10 mg/ml streptomycin). Downregulation of survivin in ZR75 cells was achieved by transfection in 6-well plates with plasmids containing shRNA directed against human survivin inserted in the vector pGFP-V-RS purchased from ORIGENE. The sequences used were: GATGGCCGAGGCTGGCTTCATCCACTGCC (902), CAGTGTTTCTTCTGCTTCAAGGAGCTGGA (903), CTTTCCTTTCTGTCAAGAAGCAGTTTGAA (904), GAGAAAGTGCGCCGTGCCATCGAGCAGCT (905) and a scrambled sequence GCACTACCAGAGCTAACTCAGATAGTACT. Once transfection efficiency reached 50% (checked by green fluorescence), cells were detached and 20-30 cells were seeded in 10 cm plates. Green-fluorescent colonies were selected and reseeded individually in 6-well plates and periodically treated with puromycin (1mg/mL). Ten clonal populations were obtained: clones 1-4, scrambled shRNA, clones 5-10, shRNA against survivin (clones 5,6: sh sequence 902; clones 7-10: sh sequence 905). **Result:** Downregulation of survivin in clones 5 and 10 significantly reduced β-catenin protein levels as compared to levels detected in clone 1 control cells.

**
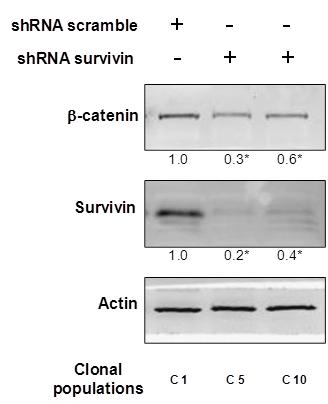
Supplementary Fig. 2. Survivin downregulation decreased β-catenin protein levels in ZR-75 breast cancer cells:** ZR-75 cells were stably transfected with shRNA directed against human survivin or a scrambled sequence. Clonal populations were obtained from green fluorescent cells: Clon 1, scrambled shRNA, clones 5 and 10, shRNA directed against human survivin. β-catenin , survivin, and actin protein levels were evaluated by western blotting. Protein levels were quantified by scanning densitometric analysis of western blots and normalized to actin. Numerical data shown are the means of results obtained in three independent experiments. Statistically significant differences compared to scrambled shRNA controls are indicated (* p<0.05).

**Supplementary information 3: Method:** HEK293T cells were transiently transfected with pEGFP-C1 or pEGFP-survivin (1.5μg) using Lipofectamine 2000 as described by the manufacturer, and treated with inhibitors. Then, 24 h later, cells were harvested, centrifuged and stored at -80°C. Upon thawing, cell extracts were prepared and submitted to western-blot analysis as described. The inhibitor bisindolylmaleimide (BIM) was from Alexis (San Diego, CA) and the inhibitor glycerocarbonate and 2-Dimethylamino-4,5,6,7-tetrabromo-1H-benzimidazole (DMAT) was from Calbiochem (San Diego, CA). **Result:** The effects of inhibiting 3 different pathways on survivin induced changes were evaluated with pharmacological inhibitors. Only the PI3K inhibitor (LY294002) suppressed the ability of survivin overexpression to increase β-catenin protein levels (Supplementary Figure 3).

**Supplementary Fig 3. PI3K inhibition suppressed survivin enhanced β-catenin protein levels:** HEK293T cells (5x10^5^) were seeded in 6-well plates and transfected with pEGFP-C1 or pEGFP-survivin (1.5 μg). After transfection, cells were treated with DMAT (CK2 inhibitor, 50 μM), BIM (PKC inhibitor, 0.5 μM) or LY294002 (PI3K inhibitor, 20 μM). After 24 h, β-catenin, survivin and actin protein levels were evaluated by western blotting. Protein levels were quantified by scanning densitometric analysis of western blots and normalized to actin. Numerical data shown are the means ± s.e.m. of results obtained in three independent experiments. Statistically significant differences compared to mock controls are indicated (*= p<0.05).
